# Light, Water, and Melatonin: The Synergistic Regulation of Phase Separation in Dementia

**DOI:** 10.3390/ijms24065835

**Published:** 2023-03-19

**Authors:** Doris Loh, Russel J. Reiter

**Affiliations:** 1 Independent Researcher, Marble Falls, TX 78654, USA; 2 Department of Cell Systems and Anatomy, UT Health San Antonio, San Antonio, TX 78229, USA

**Keywords:** melatonin, dementia, amyloid-β, ATP, adenosine, phase separation, infrared light, hydrogen bonds, viscosity, bioavailability

## Abstract

The swift rise in acceptance of molecular principles defining phase separation by a broad array of scientific disciplines is shadowed by increasing discoveries linking phase separation to pathological aggregations associated with numerous neurodegenerative disorders, including Alzheimer’s disease, that contribute to dementia. Phase separation is powered by multivalent macromolecular interactions. Importantly, the release of water molecules from protein hydration shells into bulk creates entropic gains that promote phase separation and the subsequent generation of insoluble cytotoxic aggregates that drive healthy brain cells into diseased states. Higher viscosity in interfacial waters and limited hydration in interiors of biomolecular condensates facilitate phase separation. Light, water, and melatonin constitute an ancient synergy that ensures adequate protein hydration to prevent aberrant phase separation. The 670 nm visible red wavelength found in sunlight and employed in photobiomodulation reduces interfacial and mitochondrial matrix viscosity to enhance ATP production via increasing ATP synthase motor efficiency. Melatonin is a potent antioxidant that lowers viscosity to increase ATP by scavenging excess reactive oxygen species and free radicals. Reduced viscosity by light and melatonin elevates the availability of free water molecules that allow melatonin to adopt favorable conformations that enhance intrinsic features, including binding interactions with adenosine that reinforces the adenosine moiety effect of ATP responsible for preventing water removal that causes hydrophobic collapse and aggregation in phase separation. Precise recalibration of interspecies melatonin dosages that account for differences in metabolic rates and bioavailability will ensure the efficacious reinstatement of the once-powerful ancient synergy between light, water, and melatonin in a modern world.

## 1. Introduction

Dementia is a neurodegenerative condition marked by varying levels of cognitive impairment [1], currently affecting approximately 46.8 million people around the world. It is estimated that 10 million people will develop dementia each year, and without approved pharmaceutical intervention to effectively target underlying causes [2,3,4], by the year 2050, healthcare spending attributable to dementia is projected to become a significant drain on resources representing 11–17% of total global healthcare spending [5]. Alzheimer’s disease (AD) is one of the most common causes of dementia [6,7] followed by vascular dementia (VaD) [8,9]. Together with Lewy body dementias [10] and frontotemporal dementia (FTD) [11], these major neurodegenerative disorders account for approximately 90% of all dementia cases [12]. Dysregulated aggregation of biomolecular condensates formed as a result of multivalent macromolecule interactions may underlie the common molecular mechanisms responsible for the development of all AD and non-AD dementia (nADD) [13,14,15].

An Alzheimer’s biomarker study performed within a defined population over a period of 15.7 years (maximum) found the absolute remaining lifetime risk for incident dementia to be significantly associated with elevated amyloid accumulation (hazard ratio 2.11; 95% CI 1.43–2.79). Even though 87% of the 4984 participants were diagnosed as cognitively unimpaired at enrollment, higher amyloid accumulation was a significant biomarker correlated with accelerated dementia progression [16]. Vascular risks are generally associated with the progression of VaD [8,9]. However, midlife hypertension and late-life amyloid-β (Aβ) deposition were found to be independently associated with increased dementia risk in 298 participants aged 45–64 in a study that spanned 30 years. The study was unable to identify evidence of synergy between vascular risk and Aβ deposition on a multiplicative scale in subjects with dementia, implying that unique molecular pathways may be involved in the development of dementia [17].

## 2. Aberrant Phase Separation Is the Fundamental Molecular Driver behind Dementia

In 2017, Banani et al. defined intracellular biomolecular condensates as cytosolic and nuclear micron-scale compartments not bound by membranes and formed via phase separation driven by multivalent macromolecular interactions [18]. These membraneless organelles (MLOs) are responsible for strategic cellular organization in response to changing environments including endogenous and exogenous stress [14,19,20,21]. MLOs are ubiquitously utilized not only by all eukaryotes, but also bacteria [22], and viruses which are now recognized as master architects of biomolecular condensates, using phase separation to form viral replication “factories” [23,24]. The disruption of phase separation in key cellular processes results in diseases including neurodegenerative disorders and cancer [25,26,27,28,29].

The forces that drive phase separation encompass simple density transitions in single-component fluid systems [30] to changes in macromolecule saturation levels in binary mixtures achieved via manipulating in vitro macromolecule expression levels, interaction energies, and inclusion of hydrotropes/surfactants [31,32,33,34]. Spontaneous or driven phase-separated biomolecular condensates are usually nonstoichiometric assemblies of multiple proteins and nucleic acids [35]. These multivalent macromolecules engage in site-specific interactions that conform to the “stickers-and-spacers” architecture [35,36,37], forming reversible crosslinks that may involve hydrogen bonds [38,39,40], ionic strength [41], cation-π, and π–π interactions [42] that fine-tune percolation thresholds that may further define phase separation processes [43,44,45,46].

The significant discovery by Kar et al. that fused in sarcoma (FUS) and other phase-separating RNA-binding proteins in the FET family, namely EWSR1 and TAF15, form reversible clusters of varying sizes in subsaturated solutions where phase separation was not observed [47], highlights the relevance of percolation without phase separation in phase transitions in vivo. The aggregation of FUS, EWSR1, and TAF15 are associated with neurological disorders and the three FET family RNA-binding proteins are widely expressed in most cell types [48,49]. Thus, the detection of FUS percolation clusters formed in subsaturated solutions and clusters that are coupled to phase separation in supersaturated solutions [47] offers additional insight in the aggregation of macromolecules in vivo where saturation concentration that can initiate phase separation has been questioned [50]. In this review, in order to accurately capture the concept that phase separation can be coupled to percolation as well as other phase transitions in vivo including the conversion to fibrillar solids [51,52], the term phase separation is employed in lieu of the more popular nomenclature of liquid–liquid phase separation (LLPS) which restrictively implies only viscous liquids are present in the coexisting phases [53].

### 2.1. Phase Separation of α-Synuclein into Amyloid Fibrils in Dementia

In 1992, Hardy and Higgins proposed that the deposition of amyloid fibrils in AD is the direct cause of cell loss, vascular damage, and dementia [54]. Continued research indicated that the AD disease process may be the result of the dyshomeostasis between the production and clearance of amyloid β-peptides (Aβ) [55,56]. The nomenclature committee of the International Society of Amyloidosis (ISA) defines in vivo amyloid fibrils as extracellular protein fibril deposits associated with 36 human amyloid proteins. Intracellular aggregates such as tau and α-synuclein (α-syn), which are present in all synucleinopathies and are the major component of Lewy bodies associated with Lewy body dementia and Parkinson’s disease (PD) [57,58], are excluded from this list [59,60]. However, the hallmark feature of amyloid fibrils is the self-association of soluble amyloid monomeric fibers into insoluble cross-β sheets [61,62,63], and both α-syn [64,65,66] and tau [67,68,69] have been reported to self-assemble into cross-β sheet structures.

Encoded by the *SNCA* gene on chromosome 4 [70], α-synuclein (αSyn) comprises 140 amino acids [71] with intrinsically disordered regions prone to fibrillization [72]. The aberrant self-assembly of physiological, soluble αSyn monomers into neurotoxic protein aggregates implicated in PD and other synucleinopathies [73,74] is now attributed to phase separation where macromolecular interactions trigger the irreversible liquid-to-solid transition into amyloid hydrogels containing oligomeric intermediates and cross-β-sheet fibrils [75,76,77,78,79]. The documentation of the conformational evolution of αSyn phase transitions has been successfully captured by solution and solid-state magic-angle spinning (MAS) nuclear magnetic resonance (NMR) spectroscopies [51], and the study and analysis of the material components as well as intermolecular interactions of protein molecules within αSyn condensates during phase separations were performed employing fluorescence recovery after photobleaching (FRAP) and Förster resonance energy transfer (FRET) techniques [80]. Since phase separation is an early event in αSyn aggregation, modulating phase separation and/or interfering with liquid-to-solid phase transitions during αSyn amyloid phase transitions become attractive molecular targets [81].

Phase transitions from soluble monomeric to insoluble β-sheet fibrils observed in medin employing C-direct detection NMR in combination with structural bioinformatics further supports the concept of phase separation as the common molecular pathway underlying not only AD, but also VaD [82]. Among the 36 amyloid proteins recognized by the ISA in 2018 [59], medin (AMed) is the most common amyloid found in the human body [83,84]—being an internal component of milk fat globule-EGF factor 8 (MFG-E8), also known as lactadherin—that is now associated with vascular Aβ in cerebral amyloid angiopathy (CAA) pathology [85].

Present in blood vessels of most adults over the age of 50, medin is cleaved from lactadherin to form insoluble amyloid aggregates [86,87] that co-localize with vascular Aβ deposits [88] to cause cerebrovascular dysfunction in aging mice and human subjects with VaD [89,90]. Cerebral arteriole medin is regarded as a novel biomarker for AD and VaD [91]. Even though the glycoprotein lactadherin has multiple, important physiological functions [92] including phagocytosis [93], angiogenesis [94], and mucosal repair [95], medin aggregates alter cellular homeostasis, causing microvascular endothelial dysfunction by inducing permeability via the formation of pores in lipid membranes that result in upregulated ionic current flow [96,97], a mechanism not dissimilar to how Aβ peptides form calcium ion channels in lipid bilayer membranes [98,99]. However, the conditions that trigger the cleavage of medin from lactadherin causing medin to self-assemble into pathogenic, insoluble fibrils remain unclear [82,83].

The self-recognizing aggregation of amyloid proteins is not limited to homotypic enrichment in one protein, but often also involves heterotypic interactions in condensates containing up to hundreds of proteins [100,101], and the outcome of amyloid aggregation is modified by these associated heterotypic interactions [102]. Aberrant phase separation resulting in delayed disassembly of stress granules (SGs) causes the formation of non-dynamic SGs that entrap and immobilize TAR DNA-binding protein 43 (TDP-43), rendering the protein insoluble in FTD pathogenesis [15]. Increasing understanding that phase-separating RNA-binding proteins such as FUS [47,103] and those that associate with tau [104] play important modulatory roles in the heterotypic interactions that can promote or suppress amyloid aggregation [102,105,106,107], warrants exploration of specific conditions that may trigger aberrant phase separation in RNA-binding proteins.

### 2.2. The Underappreciated Role of Hydrogen Bonds and Protein Hydration in Phase Separation in Dementia

Dementia-related neurodegenerative disorders are often associated with gene mutations that may cause the dysregulation of RNA-binding proteins responsible for the aggregation of pathological amyloid fibrils during phase separation [108]. The mechanisms reported mostly involve dysregulation in the low-complexity domains of proteins such as TDP-43 and FUS [103,105,109,110,111]. Low-complexity domains (LCDs) are generally regarded as universally disordered; however, LCDs can also adopt stable, structured conformations [112]. Therefore, aberrant phase separation observed in LCDs may involve other factors in addition to the dysregulation of intrinsically disordered regions that are essential in the promotion of phase separation. Intrinsically disordered proteins (IDPs) usually serve as necessary scaffolds that facilitate phase separation of biomolecular condensates [18,113,114,115] which can be tuned by controlling enthalpy, minimizing entropic costs in phase separation [116,117,118].

Intrinsically, phase separation is entropically unfavorable and driven predominantly by enthalpically favored protein interactions [119,120,121]. In addition to energetically favorable multivalent protein–protein interactions that offset entropic costs, variations in ions and salt concentration, pH, and temperature can result in thermodynamic changes in entropy–enthalpy compensation that regulate phase separation [122,123,124]. Phase separation in proteins such as Ddx4 [125] and hnRNPA1 [126] exhibiting upper critical solution temperature (UCST) cannot take place above a critical temperature at which the system remains homogeneous, whereas proteins exhibiting lower critical solution temperature (LCST) cannot phase separate below a critical temperature at which the system remains homogeneous [127]. Therefore, increasing temperatures can either stabilize or destabilize biomolecular condensates formed by phase separation [128], and variations in salt concentration and pH levels can further promote or disrupt phase separation [122].

Stress granules (SGs) are phase-separated membraneless organelles that are formed under endogenous and exogenous stress conditions; and persistent formation of stress granules may lead to fibrillization associated with neurodegenerative disorders [126,129]. Adjusting pH levels in solutions tunes both UCSTs and LCSTs that trigger phase separation [130,131]. Alterations in tightly controlled cytosolic pH not only affect the survival of yeast and other organisms, but also determine the material properties of phase-separated stress granule-like condensates that regulate stress responses [132,133]. A reduction in pH in yeast generates reversible condensates that dissolve upon restoration of neutral pH; whereas phase-separated condensates induced by heat in yeast can only be reversed with the help of chaperones [134]. Similarly, in lipid membranes, both pH and salt can increase or decrease critical temperatures that trigger phase separation [124].

In vitro elevation of salt concentrations produces either a dehydrating, salting-out (kosmotropic) effect that induces phase separation [135,136], or a hydrating, salting-in (chaotropic) effect that inhibits phase separation [137,138,139]. Classic interpretations of the Hofmeister effect where kosmotropic anions that remove water molecules from a protein’s hydration shell to reduce protein solubility, increasing potential for aggregation via electrostatic and hydrophobic interactions [140], and chaotropic anions that exhibit the opposite effect of increasing protein solubility, functioning as a hydrotrope preventing phase separation and aggregation [141,142] may not fully account for other relevant conditions including the reversal of the Hoffmeister effects in anions and cations [143], or the effect of pH on the aggregation of proteins relative to their isoelectric points (pI) [138]. 

At its pI of 4.7 pH, α-syn formed highly ordered, fibrillar structures even at low salt concentrations compared to other conditions due to favorable intermolecular energy interactions that compensated for the lack of salting-out effects in a low-salt environment [144]. The hydrophobic, hydrogen-bonded, Β-rich amyloid cores in α-syn are intrinsically disordered and participate in dynamic intermolecular energy interactions during fibril assembly and maturation [145,146,147]. As such, protein hydration exerts a distinct effect on the pathological aggregation of amyloid fibrils in dementia, as the hottest mutational spots are often located in residues that form protective hydrogen bonds but have lost their native protecting functions resulting in protein misfolding [148].

### 2.3. Hydration Water Activates Amyloid Aggregation and Regulates Oligomer Toxicity

The role of water hydrogen bond networks that hydrate protein surfaces in biomolecular systems is known to be active and dynamic [149,150,151,152], but its role in intracellular phase separation is often less understood. Hydrophilic residues are more hydrated than hydrophobic residues. Thus, entropy and enthalpy become the two fundamental thermodynamic driving forces in phase separation that provide the requisite energetically favorable decrease in free energy. Lum, Chandler, and Weeks postulated that the price for minimizing broken hydrogen bonds within interfacial hydration water compared to bulk is an increased enthalpic cost that scales with the surface area of the hydrophobic solute [153]. Therefore, the removal of hydration water into bulk (entropic) leads to increased protein concentration that facilitates enthalpically favored protein–protein interactions resulting in condensate formation [154,155].

In other words, desolvation or the release of water molecules from protein hydration shells into bulk water [156,157,158] create entropic gains that promote phase separation and fibril aggregation [136,159,160]. Tau proteins that phase separate from salting-out effects via increased salt concentration become dehydrated and mature into irreversible, canonical tau fibrils, whereas tau proteins in reversible condensates formed via electrostatically driven phase separation remain hydrated and do not mature into pathogenic fibrils with restricted water accessibility and increased micro-viscosity [135]. Mutational hotspots with structural defects that affect protein interactions in monomeric states can be regions with an immense propensity to aggregate if the exclusion or removal of water in those regions confer a high thermodynamic benefit [148].

In 1959, Walter Kauzmann proposed that hydrophobicity in protein hydration shells drives protein folding where protein hydration accumulates hydrophobic free energy and removing the water molecule from the hydration shell can supply the free energy required to drive protein folding [161]. This hypothesis remained largely controversial [162,163] until support from experimental evidence on protein hydration shells was published. When the original clathrate water hydration shell used by Kauzmann in 1959 was replaced by a dynamic one formed by van der Waals (vdW) attraction [164], it became clear that the structural differences between water molecules in hydration shells and bulk [165] contributed to changes in free energy produced in vdW attraction interactions that favored protein folding [166,167]. Furthermore, the fact that the addition of salt can tune the hydrophobic effect by breaking hydrogen bonds in hydration shells [168] and rearrange the hydrogen-bonding environment in interfacial waters [169], provides additional support for the role of dehydration in the formation of pathogenic amyloid fibrils.

Highly sensitive femtosecond time-resolved fluorescence spectroscopy revealed the presence of dynamically distinct, confined interfacial hydration water molecules with severely restrained mobility compared to bulk water [170]. The removal of these confined water molecules in the intrinsically disordered amyloidogenic NAC domain of a-syn changes the rate of intramolecular backbone reconfiguration to facilitate the formation of cytotoxic oligomers [171] via intermolecular associations involving chain desolvation, indicating the entropically favored removal of confined water molecules into bulk water [170]. Early studies found the aggregation of protofilaments from Aβ16-22 peptides was due to the hydrophobic collapse of protofilaments caused by water molecules being released [172,173]. Similarly, the aggregation propensity of Aβ1-40 was significantly elevated via escalating salt concentrations to enhance salting-out effects, with the implication of heightened protein–protein interaction energy and diminished hydrogen-bond strength [174,175]. Out of 3.45 hydrogen bonds formed by a water molecule, only 2.41 are considered “strong” hydrogen bonds. Per the hydrophobic effect, the ability to form hydrogen bonds directly affects the stability of protein where net stabilization at 1–2 kcal/mol can be provided by each intramolecular hydrogen bond [176].

Limited hydration in the interior of MLOs fosters a favorable environment for liquid-to-solid phase transitions observed in amyloidogenic aggregates that are often preceded by liquid-to-liquid phase separation [79,177]. During α-syn nucleation, limited hydration lowers the desolvation barrier and intermolecular hydrogen bond barrier. Thus, the simple removal of confined water molecules in the hydrophobic amyloid NAC domain in α-syn can easily breach high desolvation barriers that normally prevent aggregation of amyloid fibrils [178,179,180]. Furthermore, the level of protein hydration determines whether homogeneous or heterogeneous nucleation is selected as the primary aggregation mechanism, which further defines the type of amyloid polymorph generated as well as the cytotoxicity of the α-syn oligomers formed [178]. Unfortunately, reduced hydration may be an inevitable phenomenon associated with aging in the human brain.

During normal aging, even though total protein content in the normal aging brain can decline by 5–15% between the ages of 30 and 90 years, water-soluble protein content actually increases by 16–48%, providing a viable explanation for observations of significantly decreased water content in normal aging brain cells [181,182]. The fact that confined and “bridging” interfacial water molecules have limited mobility and exceptionally slow hydrogen-bond rearrangement compared to bulk water, respectively, [170,183] highlights the importance of the binding dynamics of interfacial hydration water around residues located in IDPs prone to phase separation under conditions of limited mobility and hydration [184,185]. Atomistic MD simulations revealed that during the growth of Aβ_9-40_ fibrils, the collective movement of confined interfacial water with reduced mobility provides the entropic energy for pathogenic fibril formation via the removal of 60–85 water molecules that concurrently supplies a dry binding interface between filament and monomer [186]. Consequently, the ability to manipulate the relative thermodynamics of hydrogen bonds [187] in interfacial water compared to bulk becomes an extremely attractive proposition in the regulation of protein aggregation in dementia.

### 2.4. The Synergistic Regulation of Hydrogen Bonds by Light, Water, and Melatonin

The International Union of Pure and Applied Chemistry (IUPAC) defined a hydrogen bond as “an attractive interaction between a hydrogen atom from a molecule or a molecular fragment X–H in which X is more electronegative than H, and an atom or a group of atoms in the same or a different molecule, in which there is evidence of bond formation” [188]. Although hydrogen bonding can affect important physicochemical properties including density, refractive index, and conductivity [189], due to a limitation of scope, this review is solely focused on the relevant associations between hydrogen bonds and viscosity [190,191] in the context of protein hydration in phase separation in dementia.

Interfacial water can exhibit viscosity 106 times higher than bulk water [192], and the breaking and forming of hydrogen bonds in water [193] can affect viscosity of interfacial water. Viscosity is measured in units of centipoises (cPs) [194], and viscosity can accurately indicate flow resistance in water and other solvents. Viscosity in interfacial water is increased by hydrophilicity and reduced by hydrophobicity, implying the strength of the hydrogen bond is critical to maintaining the integrity of the viscous phase in interfacial water [192]. Low-level microwaves, and other electric and electromagnetic fields (EMF) can restructure hydrogen bonding [195,196] where weakened or broken hydrogen bonds decrease viscosity [197,198] and the formation of stronger hydrogen bonds increases absolute viscosity [199]. Light is a form of electromagnetic radiation (EMR) [200], and plants are exposed to an entire spectrum of EMR from sunlight. However, plants only absorb visible light but reflect infrared light.

During canopy photosynthesis, visible light from the sun is absorbed and utilized while a directly proportional amount of infrared is reflected [201,202,203]. A tight, linear correlation exists between canopy photosynthesis and correspondingly reflected NIR in all types of plants examined, including well-watered crops, wetland vegetation, grasslands, and savannas, with different functions, structure, capacity, and even, soil conditions [204]. Surprisingly, or not, a higher level of greenness or presence of vegetation is associated with reduced risk for AD (20%, odds ratio 0.80; 95% CI, 0.75–0.85) and non-AD dementia (11%, odds ratio, 0.89; 95% CI, 0.82–0.96) [205]. However, subjects with dementia treated with UVB irradiation did not exhibit any of the greenness effect even though plants are exposed to both spectrums in sunlight [206].

Similarly, photobiomodulation employing visible red light (670 nm), non-visible near infrared (NIR, 800–1090 nm), and even far infrared (FIR, 3–25 µm) show encouraging results in the attenuation of symptoms associated with dementia including a reduction in Aβ deposition, size and number of plaque and fibril formation, clearance of misfolded proteins, increased ATP production and reduced ROS production, improved executive and cognitive functions, processing speed, memory performance, mood, energy, and sleep [207,208,209,210,211,212,213,214,215,216,217] (Table 1). The proposal that red and near-infrared wavelengths may promote melatonin synthesis in mitochondria via the pathway involving nitric oxide and enhanced activity of soluble adenylyl cyclase further bolsters the synergistic relationship between light and melatonin [218,219].

**Figure 1:**
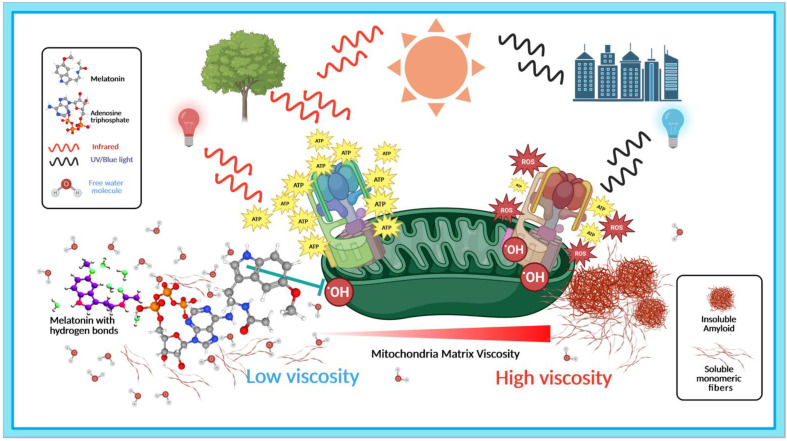
Visible 670 nm red light reduces viscosity in mitochondria interfacial water to increase free water molecules and enhance ATP synthase ability to generate more adenosine triphosphate (ATP). Reactive oxygen species (ROS) increase viscosity and lower ATP synthase efficiency to inhibit ATP production. Melatonin lowers viscosity by scavenging hydroxyl radical (^•^OH) and ROS. Increased free water molecules from lower viscosity form stronger hydrogen bonds with melatonin to enhance its intrinsic features that include binding interactions with the adenosine moiety of ATP, inhibiting water removal from protein hydration shells that facilitate amyloid fibril aggregation and solubilizing aggregates formed as the result of aberrant phase separation.

The ability to increase adenosine triphosphate (ATP) production in mitochondria is one of the most widely accepted mechanisms behind the effectiveness of photobiomodulation in dementia and other health challenges [211,217,220,221,222]. The fact that both infrared light and melatonin increase ATP production, and the adenosine moiety of ATP which is structurally similar to melatonin is capable of solubilizing protein aggregation point to the existence of a most unexpected, dynamic relationship between NIR light and melatonin that is inextricably connected to the regulation of hydrogen bonds, viscosity, protein hydration, and protein aggregation (Figure 1). The following section will present what is currently known about molecular mechanisms that drive the synergistic relationships between light, water, and melatonin in the regulation of phase separation of pathological aggregates in dementia. In subsequent discussions, the term light refers to red and near-infrared wavelengths unless otherwise indicated.

## 3. Light, Water, and Melatonin: Ancient Synergies in a Modern World

The synergistic relationship between melatonin, water, and light may have originated billions of years ago when primitive unicellular organisms depended on this effective and precise synergy to modulate phase separation to control protein aggregation and associated biological effects. The efficacy of this synergy also provides a credible explanation for the immensely successful and rapid distribution of melatonin via horizontal gene transfer [223]. The discovery of the serotonin N-acetyltransferase (*SNAT*) gene responsible for the synthesis of essential melatonin substrate N-acetylserotonin (NAS) in archaea [224,225] firmly establishes the quintessential role played by melatonin in early primitive organisms that use phase separation as the fundamental driver for relevant biochemical and biophysical processes to support metabolism, replication, and survival [226,227,228,229,230,231,232].

Melatonin (N-acetyl-5-methoxytryptamine) was first isolated from bovine pineal gland in 1958 [233]. Since then, revelations from the study of melatonin led to a continuously expanding list of appellations that aim to describe its impressive yet often pleiotropic and contradictory behaviors. Melatonin is known as a hormone, an antioxidant, an anticancer agent, an antiviral, an autocoid, a chronobiotic, a hypnotic, an anxiolytic, a glycolytic, a sleep aid, a universal panacea, a biological modifier, and even a Higgs boson [234]. These nomenclatures are excellent illustrations of some of the broad-based metabolic effects achieved by melatonin as it regulates fundamental phase separation processes in living organisms. The role of melatonin in the regulation of phase separation in the context of neurodegenerative disorders, cancer multidrug resistance, and viral phase separation are clearly defined in several in-depth reviews [230,235,236]. Due to a limitation of scope, the reader may review these extensive discussions for a better understanding of molecular mechanisms employed by melatonin in the regulation of phase separation under different biological contexts. This review will focus on the presentation of known, relevant molecular mechanisms that facilitate and enhance the synergistic relationship between light, water, and melatonin in the regulation of phase separation in dementia.

### 3.1. Light, Water, and Melatonin: Viscous Relationships with Hydrogen Bonds

Water molecules confined in interfacial hydration water exhibit severely restrained mobility compared to bulk water [170]. The mobility of these water molecules is reduced by interfacial viscosities as high as 106 times that of bulk [192,237]. However, the viscosity of water constrained in extremely narrow spaces such as the interior of carbon nanotubes increases and decreases with increased and decreased diameters, respectively [238,239]. In carbon nanotubes with diameters below 20 Å, water stops behaving like bulk water with different boiling points, self-diffusion coefficient, and viscosity [238,240,241,242]. Even the mobility of water molecules in ultra-confined spaces is enhanced by reduced viscosity [239,243] which is facilitated by a reduction in hydrogen bonds.

In general, viscosity is increased by stronger intermolecular interactions that form more hydrogen bonds in water molecules [238]. During phase separation, the variation in internal micro-viscosity between tau droplets formed via homotypic and heterotypic associations can be as much as a 7-fold increase [244]. Systematic reductions in droplet micro-viscosity during biological aging may imply continuously evolving intermolecular interactions that shift droplet equilibrium, modifying aggregation potential that favor pathological outcomes [14,245,246]. Therefore, novel properties such as enhanced solubility, diffusion, and electron transfer in specially treated water molecules with lower viscosity and reduced/broken hydrogen bonds [247] may have distinctive effects on the modulation of aberrant protein aggregation in dementia.

Hydrogen bonds (HBs) can be reduced/broken by hot electron transfer when plain, deionized bulk water is allowed to flow through gold nanoparticles under resonant illumination. The water—known as plasmon-activated water (PAW)—created by this method exhibits features conspicuously different from bulk even at room temperature [247]. The reduced intermolecular hydrogen bonds in water molecules not only decrease viscosity, but also impart a higher degree of freedom in interaction that allows the formation of stronger intermolecular hydrogen bonding with hydrophilic solutes while enhancing the solubility of hydrophobic solutes [247,248]. Essentially, the elevated interactions with other molecules via increased free water molecules in PAW enhance the intrinsic activities of these molecules. Melatonin is known to dissolve poorly in water [249]; however, melatonin is able to form stronger hydrogen bonds in PAW resulting in enhancement of solubility between ~120% [248] to ~150% [250].

#### 3.1.1. PAW Modulates Melatonin Hydrogen Bonding and Conformation

Melatonin has five distinct hydrogen bonding sites for water, forming up to five hydrogen bonds with water molecules simultaneously at varying strengths. Two of these hydrogen molecules from two water molecules can even reside indefinitely when they are coordinated with the O of the amide group due to the high degree of stability between the H-bond as indicated by Helmholtz free energy [251,252]. For melatonin, water can either be a H-bond donor or acceptor, depending on the site it is attached to. However, even one single water molecule attached to melatonin can change its conformational preference by modulating the relative energies of the conformations and the heights of the barriers that separate conformations, where strong H-bonds can produce substantial electronic frequency shifts. Furthermore, the relative abundance of the conformations can also be regulated by H-bonds, implying that preferential binding between specific sites and water molecules can produce conformational clusters with populations as high as 10 times over other species [252]. In bulk water, melatonin forms the strongest H-bond with its carbonyl O group, stabilizing its tendency to self-aggregate resulting in low solubility [253].

Melatonin prepared in PAW compared to bulk deionized water exhibited enhanced clearance of hydroxyl radical at 11.9% vs. 6.69%, respectively; its antiviral potency against dengue virus in infected human hepatocarcinoma cells is also enhanced, reducing infectivity by 14.7% vs. 20.6% in bulk [250]. Male Wistar rats subjected to chronic sleep deprivation (CSD) using the disc-on-water methodology [254] and treated with 10 mg/kg melatonin via intraperitoneal (IP) injection dissolved in PAW exhibited significantly better results in all parameters detected, including hepatic function and metabolic activity, than control (no treatment), CSD only, and CSD + melatonin dissolved in bulk deionized water groups [250]. It is plausible that when melatonin is dissolved in PAW, the intrinsic anti-inflammatory properties of PAW may also be responsible for molecular mechanisms that support/enhance melatonin’s antiviral and antioxidative features. Indeed, APP/PS1 transgenic AD mice treated with PAW showed improved memory function and reduced amyloid burden, potentially via anti-inflammatory and anti-oxidative effects, compared to age-matched wild-type controls [255,256]. There is no doubt that the anti-oxidative properties of PAW enhance melatonin’s intrinsic activities. However, the molecular mechanism involved is an unexpected, viscous one.

#### 3.1.2. Reactive Oxygen Species Increase Viscosity

Hydrophilicity enhances viscosity in interfacial water at values up to ~106 times that of bulk due to an increase in ordering and hydrogen-bond dynamics [192]. The negative polarity of reactive oxygen species (ROS) is able to increase hydrophilicity and elevate viscosity. When the oxygen atom of one of the most reactive ROS hydroxyl radical (^•^OH) becomes highly negative and acts as a hydrogen bond acceptor, it can lower the reaction barrier stabilizing ^•^OH bonding to water during the polar transition state. Thus, water and viscosity of water can modulate and stabilize the highly reactive ^•^OH [257]. In bulk water, ^•^OH forms three stable hydrogen bonds and a weaker hemibond with surrounding water molecules comprising its solvation shell [258].

In mitochondria, ^•^OH is derived from superoxide radicals produced as a result of a one-electron reduction of oxygen (O_2_) from electron leakage during mitochondrial electron transport [259,260]. Simply stated, the presence of excess, unneutralized ROS can significantly elevate viscosity in these essential energy-producing organelles, negatively impacting mitochondrial functions and ATP production associated with pathological Aβ aggregation [261]. Hydrogen peroxide (H_2_O_2_)—a ubiquitous ROS with classical intracellular signaling functions at lower physiological levels [262]—is also produced in mitochondria from electrons leaked during mitochondrial electron transport activities [259]. Similar to ^•^OH, H_2_O_2_ accumulation can increase matrix viscosity in mitochondria [263,264]. Furthermore, an NIR emissive fluorescent probe with a large Stokes shift detected significantly elevated viscosity and H_2_O_2_ levels in brain mitochondria of APP/PS1 transgenic AD mice compared to normal BALB/c mice [265].

#### 3.1.3. Reduction in Viscosity and Hydrogen Bonds Enhance Melatonin ROS Scavenging

Melatonin is known for its ability to scavenge ^•^OH and other free radicals [266,267,268,269,270] where one molecule of melatonin can scavenge two ^•^OH radicals to produce the stable cyclic 3-hydroxymelatonin (3-OHM) metabolite [266]. However, the addition of only one water molecule that provides an H-bonding relay pathway significantly lowered the energy barrier in the tautomerization step to enhance the scavenging potential by melatonin [271]. The fact that melatonin prepared in PAW exhibit 78% increased effectiveness in ^•^OH scavenging compared to bulk (11.9% vs. 6.69%) [250] implies that melatonin may adopt more favorable conformations that enhance its intrinsic activities as a result of stronger H-bonds formed in water with reduced viscosity and H-bonds compared to bulk.

In the context of aberrant protein aggregation in dementia, the signature reduction in viscosity and H-bonds in PAW inadvertently accentuates an unconventional but relevant perspective on the viscous relationships between light, melatonin, and ROS that surprisingly, or not, converge on the synthesis of ATP in mitochondria. In response to conditions that reduce ATP, budding yeast conserves energy by increasing cytosolic viscosity to slow cellular processes by reducing protein diffusion rates. Additionally, increased viscosity modulates phase separation, impeding the formation of stress granules and inducing aberrant phase separation to form aggregates that were not present in cells that could not elevate viscosity [272].

### 3.2. Light, Melatonin, and Viscosity in the Elevation of ATP Synthesis

Mitochondrial matrix exists mostly as interfacial water due to the density of proteins, and matrix water exhibits similar restrained mobility as interfacial water. Consequently, matrix water is significantly more viscous than cytoplasm [273]. The viscosity of the mitochondrial matrix is correlated with the respiratory state of the organelle that can affect not only signal transduction, but also how mitochondrial networks are organized. The abnormal elevation of viscosity in mitochondria results in dysregulation in metabolite diffusion that can cause aberrant phase separation resulting in malignancies associated with fatty liver, diabetes, atherosclerosis, accelerated aging, cancer, AD, and other neurodegenerative disorders. Therefore, the accurate detection and determination of mitochondrial viscosity can facilitate the understanding of molecular mechanisms behind various diseases associated with mitochondrial dysfunctions [265,274,275,276,277,278,279,280,281,282,283,284,285,286,287,288,289,290,291,292,293,294]. A fluorescent probe that can detect mitochondrial viscosity fluctuations was used for the first time in the successful, early diagnosis of liver and kidney injury in animal models [295], while other probes are employed to effectively distinguish normal cells from cancerous cells with distinct, elevated viscosity [296,297,298,299].

In HeLa cells, the average viscosity of mitochondria is determined to be ~62.8 cP [300], in stark contrast to the 2.04 +/− 0.49 cP obtained for HeLa nucleoplasm viscosity which is already higher than that in HeLa cytoplasm [301]. Furthermore, treatment with pharmaceuticals, such as monensin and nystatin, can further drive matrix viscosity up to 90.5 and 109 cP, respectively [300,302]. Mitochondria of HeLa cells under oxidative stress generate a tremendous amount of ROS [303]. Therefore, ROS such as ^•^OH, which is naturally produced during mitochondrial respiration, and excess oxidative stress, can potentially increase matrix viscosity from its hydrogen-bonding interactions with water molecules. Dual-targeting fluorescent probes are developed to easily identify viscosity changes in mitochondria in the presence of specific free radical species [304]. Mechanistically, increased viscosity in the matrix can result in the lower production of ATP catalyzed by the ATP synthase.

#### 3.2.1. Efficiency of ATP Synthase Is Modulated by Viscosity

The mitochondrial ATP synthase (F_0_F_1_) is a rotary motor enzyme with a proton-driven F_0_ motor that is embedded in the inner mitochondrial membrane and is connected to the ATP-driven F_1_ motor that protrudes into the mitochondrial matrix [305,306,307]. The higher viscosity of the medium can slow down the rotation of the F_1_ motor to reduce ATP synthesis not only in mitochondria [308] but also chloroplasts [309]. While ATPase turnover rates are more effective when detected by probes designed with lower viscous drag [310], viscous drag can dramatically slow the rate of rotation to 3% of the enzyme turnover rate in *Escherichia coli* [311].

Nonetheless, 100% efficiency of the F_1_ rotor can theoretically be achieved if the 120° power strokes rotate at a constant angular velocity [312]. However, power stroke and dwell duration are easily modified by viscosity. Viscous loads applied to the ATP F_1_ motor of *E. coli* can cause the increase in the duration of the 120° power stroke that is correlated to a 20-fold increase in the length of the dwell. Thus, the power stroke velocity is limited by the viscous load on the motor, and consequently, increases in transition time are the direct result of increases in viscosity and not from inhibition of the ATPase by other means [313]. A deeper analysis of viscosity sensitivity showed that viscous drag on rotations of the γ-subunit in the F_1_ motor [314] can cause variations of more than 5000-fold by using a variety of rotation probes [315].

#### 3.2.2. The 670 nm Wavelength Elevates ATP Production in Mitochondria

The benefits of photobiomodulation employing the 670 nm wavelength for dementia and other neurodegenerative disorders are extensively documented (Table 1). Even though improved ATP production and reduced ROS production are associated with the use of 670 nm irradiation, the exact mechanism responsible for these effects remains controversial. Experimental works employing 670 nm report the reduction in inflammation via increased expression of cytochrome C oxidase (COX) in an age-related macular degeneration mouse model [316]; increased COX expression and ATPase activities in Wistar rats exposed to suppressive effects of fluorescent light [317]; significantly elevated ATP production in aging mouse retina via increased COX expression [318]; and the restoration of neuronal ATP and prevention of apoptosis induced by potassium cyanide—an irreversible inhibitor of COX [319]. Therefore, benefits from photobiomodulation, especially the enhancement of ATP production and mitochondrial function, are generally believed to be associated with the involvement of COX via increased COX expression and activities.

COX, or complex IV [320], is the fourth enzyme that catalyzes the transfer of electrons from ferricytochrome C to oxygen in the mitochondrial electron-transport complexes, and COX is highly susceptible to inactivation by oxidative damage induced by ROS including ^•^OH and 4-hydroxynonenal (HNE), a major lipid peroxidation product [321,322,323,324,325]. Even though COX is viewed as the primary photoacceptor, molecular mechanism that elucidates the association of irradiation by 710–790 nm and 650–680 nm wavelengths with reduced and oxidized states of COX, respectively, remain elusive [326]. Furthermore, experimental work that combined nanoindentation and 670 nm laser irradiation to modulate viscosities of interfacial water supports the proposal that lower viscosity in mitochondria is the real driver behind photobiomodulation propelling enhanced ATP synthesis, and not increased COX expression and activities [327,328,329]. However, if reduced viscosity from light irradiation is responsible for increased ATP synthesis via increased power stroke velocity producing more efficient F_1_ motor rotations, then this proposal should be inclusive of COX involvement also.

#### 3.2.3. Viscosity Modulates COX Activities in Mitochondria

In 1987, the main activity of COX—the oxidation of ferricytochrome C by COX—was demonstrated to be viscosity-dependent at both high and low ionic strengths [330]. While laser flash photolysis revealed a dramatic decrease in the rate of intramolecular electron transfer (IET) between the heme and molybdenum centers of chicken liver sulfite oxidase when solution viscosity was increased [331]. The evidence supporting the enhancement of ATP synthesis via light irradiation is solid [332,333], and it is also not unreasonable to propose that the reduction in mitochondrial matrix viscosity by light or ROS scavenging can increase ATP production, and increased ATP is associated with clearance of pathogenic aggregates from aberrant phase separation.

Thus, the ability to clear Aβ aggregation by the antioxidant epigallocatechin-3-gallate (EGCG) may be the result of upregulated COX activities and ATP production from reduced ROS and matrix viscosity [334,335]. In human neuroblastoma (SH-EP) cells, 670 nm irradiation dramatically elevated ATP levels by 20% which was subsequently diminished after irradiation-associated clearance of Aβ42 aggregation. Both 670 nm irradiation and EGCG were independently able to reduce Aβ42 aggregation at the expense of ATP consumption compared to controls. However, the combined, complementary treatment produced even better results in the clearance of amyloid aggregates compared to controls [334].

### 3.3. Melatonin Prevents and Disaggregates Aberrant Protein Aggregation in Dementia via Association with ATP

Melatonin, a mitochondria-targeted molecule [336] that is known for being a potent ROS scavenger [266,267,268,269,270,271], promotes ATP synthesis via the elevation of COX expression and activities. Melatonin administered to aged rats at 10 mg/kg per day in drinking water prevented the 30% age-related decline in COX activity while abolishing concomitant elevation of H_2_O_2_ in brain mitochondria of aged rats compared to controls [337]. Melatonin administered orally at 10 mg/kg/day for 17 weeks to male Zücker diabetic fatty (ZDF) rats restored the 25% decline in renal mitochondrial COX activity and attenuated other mitochondrial dysfunctions including diminished ATP production compared to lean controls [338]. Melatonin administered in drinking water at the same amount to ZDF rats reversed the 76% decline in brown adipose tissue mitochondria COX activity by 35% while increasing COX activity by a staggering 31% in normal, lean controls [339].

#### 3.3.1. Melatonin Elevates ATP Production via Modulation of COX and Viscosity

Even though cyanide (CN^−^)—a highly cytotoxic molecule that inhibits COX to suppress mitochondrial respiration and ATP production, and elevates ROS by modulating antioxidant defense—is proposed to be a novel mammalian gasotransmitter that can stimulate COX activity and enhance cellular bioenergetics at low endogenous nanomolar levels, at levels beyond 10 μM, CN^−^ remains exceedingly toxic [340,341,342,343,344,345]. As a result, sophisticated dual-response sensors and probes are used to detect fluctuations in mitochondria viscosity in the presence of varying levels of cyanide in living cells [278,346].

Not unexpectedly, the in vitro study of rat brain mitochondria treated with 5 µM potassium cyanide revealed that 50% inhibition of COX activity was nearly entirely counteracted by treatment with 100 µM melatonin in a dose-dependent manner compared to control; while COX activity in rat liver mitochondria under same treatment conditions achieved 30% higher efficiency than control. However, at 100 µM cyanide exposure, even 5 mM of melatonin was unable to reverse the 100% inactivation of COX [347]. In vivo administration of melatonin at 10 mg/kg (IP) significantly elevated COX activity in rat brain and liver mitochondria in a time-dependent manner while reversing COX activity inhibition and preventing mitochondrial damage and oxidative stress induced by ruthenium red treatment at 60 µg/kg (IP) [348]. Ruthenium complexes can also increase viscosity and induce cell apoptosis via ROS-mediated mitochondrial pathways [349,350].

#### 3.3.2. Fibril Disaggregation by Melatonin Is Dose-Dependent

Melatonin is intensively studied and extensively reviewed as a likely ideal therapeutic molecule for AD and other neurodegenerative disorders [351,352,353,354,355]. A novel understanding of melatonin regulation of biomolecular condensate phase separation in neurodegenerative disorders [230] provides additional relevant molecular mechanisms behind reported observation including the inhibition, destabilization, reduction, and delay of α-Syn and Aβ fibril aggregation. Melatonin not only increased survival rates in transgenic AD mice, but also reversed Aβ-induced synaptic disorder, memory deficit, neurodegeneration, as well as phosphorylation of tau in wild-type mice injected with Aβ peptides [356,357,358,359,360,361,362,363,364,365,366] (Table 2). However, inconsistent results were observed when there were discrepancies in dosage and timing/duration of administration [363].

Similarly, in vitro studies on tau fibril aggregation and disaggregation in the presence of melatonin at varying strengths found disaggregation effects to be dose-dependent where 100 μM led to 14% disaggregation while 5000 μM disaggregated ~54% of pre-formed repeat domain tau [367] (Table 2). However, 200 μM melatonin treatment in full-length tau aggregates failed to produce morphological changes, and 5000 μM treatment could not prevent aggregation but was able to disaggregate tau fibrils into small, broken filaments [368] (Table 2).

#### 3.3.3. Melatonin Hydrogen Bonding May Modulate Salt Bridge Formation in Aggregates

The general consensus on the disaggregation mechanism employed by melatonin is the disruption of salt bridge formation or the reduction of hydrophobic interaction between proteins [358,359] (Table 2). Salt bridges formed between tau proteins can strengthen and stabilize the core of the paired helical filaments which enhances aggregation [367,369]. Both hydrogen bonds and salt bridges provide favorable free energy during protein–protein binding. Therefore, unfulfilled hydrogen bonds or isolated charges without forming salt bridges can destabilize binding due to the desolvation effect [370,371].

During Aβ oligomerization, the prerequisite expulsion of water molecules from protein hydration shells facilitates the formation of salt bridges [372]. In general, weaker hydrogen bonds are formed in interfacial regions due to the restrictive translational and rotational freedom constraints in interfacial regions. Consequently, more water molecules are required in interfacial regions for bridging hydrogen bond networks across protein interfaces [370]. When interacting with melatonin, water can act as both a H-bond donor to the amide carbonyl, methoxy oxygen, or indole π clouds and a H-bond acceptor from the amide NH and indole NH groups [252]. Therefore, the ability to form π hydrogen bonds [373] potentially allows melatonin to prevent the formation of salt bridges that impede intramolecular tau filament aggregation [374]. However, in vitro studies produced results that did not fully support in vivo and ex vivo work on melatonin and tau hyperphosphorylation [367,368] (Table 2).

#### 3.3.4. Hyperphosphorylation Reduces Water Hydration during Fibril Aggregation

Hyperphosphorylation of tau is a reversible physiological process, but abnormal hyperphosphorylation in neurodegenerative disorders including AD is resistant to dephosphorylation and proteolysis [375,376,377,378]. It is believed that the cytotoxicity of Aβ is tau-dependent where tau and Aβ together drive healthy neurons into diseased states and that both Aβ and tau toxicity reinforce each other via a feedback loop [379,380,381,382]. The oligomerization of tau fibrils resulting in the formation of pathological tau aggregates is thermodynamically facilitated by hyperphosphorylation of tau proteins [383]. Hydrophobically driven phase separation which leads to the removal of water molecules from protein hydration shells is the predominant interaction that amplifies hydrophobic attractions that cause hyperphosphorylation of tau and fibrillization [135,384]. Recall tau proteins that phase separate due to salting-out effects mature into pathogenic, irreversible, canonical tau fibrils with restricted water accessibility and increased micro-viscosity [135] (see Section 2.3).

Furthermore, hyperphosphorylation can generate conformation changes critical for in vitro phase separation of full-length tau which precedes aggregation. Hyperphosphorylation shifts the equilibrium between soluble and phase-separated tau to favor the droplet state, enhancing maturation that initiates pathological aggregation [385]. Consequently, the ability to form hydrogen bonds to maintain protein solubility may determine the level of effectiveness of melatonin treatment in the prevention of tau hyperphosphorylation and subsequent phase separation events that ultimately result in the formation of pathological tau aggregates.

Therefore, in vitro work that showed dose-dependent disaggregation of pre-formed tau fibrils but the inability to prevent aggregation even at high concentrations of 5000 μM in contrast to in vivo and ex vivo work that reported a significant reduction in tau hyperphosphorylation even after the establishment of tauopathy (Table 2) may simply reflect the absence of ATP that can modulate hydrophobic interactions from hydrogen-bonding activities. In the context of phase separation in dementia, ATP may be the quintessential lynchpin that brings light, water, and melatonin together in a dynamic and effective synergy. After all, the regulation of aberrant protein aggregation in dementia by light and melatonin is associated with molecular mechanisms including reduced viscosity, hydrogen bonding, protein hydration, and elevation of ATP synthesis (Figure 1).

### 3.4. Light, Water, and Melatonin: The Adenosine Moiety Effect of ATP

The ability of ATP to solubilize hydrophobic substances in aqueous solutions at neutral and elevated pH was first reported by Mandl and Neuberg in 1952 [386]. Several decades later, ATP was observed to behave as a hydrotrope, solubilizing and dissolving protein aggregates in Xenopus oocyte nucleoli, preventing the aggregation of synthetic Aβ_42_ peptides, and even dissolving preformed tau fibrils [387,388]. However, employing all-atom molecular dynamics (MD) simulations, Kurisaki et al. observed contradictory results where ATP actually did not have any effect on the dissociation of monomers or the decomposition of the Aβ_42_ oligomer. Instead, the hydrophobic adenosine moiety of ATP was reported to dissociate Aβ_42_ monomers via contacts with Aβ_42_ backbone atoms, potentially dissolving the Aβ_42_ oligomer by shifting thermal equilibrium from an on-pathway species to an off-pathway species [389].

These observations were further clarified by Mehringer et al. demonstrating via MD simulations that ATP did not exhibit classic features of a hydrotrope or displayed chaotropic salting-in effects. In fact, ATP can be considered a kosmotropic anion with salting-out effects as a result of the triphosphate moiety of ATP capable of lowering the solubility of organic compounds in water. The ability of ATP to prevent and dissolve aggregates formed by phase separation observed in earlier works [387,388] is facilitated mainly by the interaction of the aromatic adenosine moiety in ATP with intrinsically disordered proteins, while the highly charged phosphate moiety served to heighten the solubility of the hydrophobic adenosine in ATP [390]. This molecular mechanism clearly explains why AD transgenic mice exhibit significantly reduced ATP production and mitochondrial dysfunction [391]. The adenosine moiety prevents hydrophobic collapse and aggregation by increasing solubility that prevents water removal [173]. As a consequence, the presence of ATP is highly effective in the suppression of Aβ_16-22_ peptide aggregation [392].

Adenosine is a primordial metabolite [393] that is an integral component of ATP and RNA [394,395]. Not unexpectedly, both ATP and RNA modulate phase separation biphasically where low concentrations enhance phase separation but high concentrations inhibit droplet formation [387,396,397,398,399]. MD simulations demonstrate succinctly that the dissolution of FUS by ATP-Mg^2+^ is promoted by solubilization via the adenine moiety and the phosphate moiety served only to enhance the requisite hydration effect [400].

**Figure 2:**
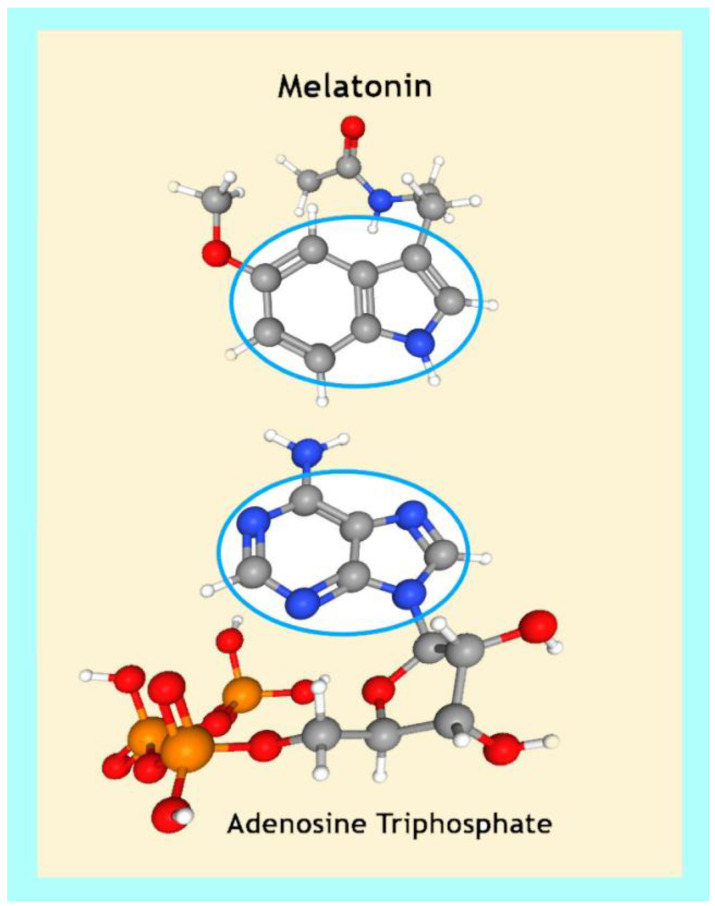
Homologous molecular structures between the electron-rich aromatic indole moiety in the melatonin molecule [427] and the adenosine moiety of ATP [428,429].

Mechanistically, the adenosine moiety may prevent amyloid fibril formation by interfering with Aβ peptide π–π stacking [401,402]. Interestingly, the indole ring of tested indole derivatives effectively inhibited the formation of amyloid fibrils in hen egg-white lysozyme induced by low pH and high temperatures via hydrophobic interactions that accelerated disaggregation and destabilized the amyloid fibrillar state [403,404]. Therefore, it is perhaps not an evolutionary coincidence that melatonin not only exhibits structural homology to the adenosine moiety of ATP [401] (Figure 2), but also binds to adenosine via a hydrogen bond [405,406,407,408]. Consequently, ATP and melatonin may have been used for billions of years by living organisms to efficiently regulate phase separation in proteins with a high propensity for aggregation [230,401,409].

Arguably, the absence of ATP, despite the ability of melatonin to disrupt salt bridge formation, may be the reason why in vitro works on melatonin and tau fibril aggregation reported distinctly different results in the inhibition of fibril formation that could not confirm in vivo and ex vivo observations even at high concentrations of 5000 µM (Table 2).

Extracellular adenosine is derived from the degradation of ATP and adenosine monophosphate (AMP), whereas hydrolysis of AMP is the main source of intracellular adenosine [410,411]. It is estimated that extracellular adenosine can rise 1000-fold from the low nanomolar range of ~20–300 nM to the low micromolar range as high as 30 µM under conditions of high physical stress including extreme exercise and high altitude with low ambient oxygen [412]. Neurodegenerative diseases, inflammatory conditions, autoimmune diseases, cancer, diabetes, and cerebral ischaemia are pathological conditions associated with elevated extracellular adenosine [410,413,414,415,416,417].

Under optimal conditions, the high reserve/maximum capacity of melatonin synthesis in humans theoretically confers enhanced survival fitness as higher melatonin production allows rapid adaptation to unpredicted internal and external stressors [418]. Assuming that melatonin can be bound to adenosine at a 1:4 ratio [406,407,408], 6–20 nM plasma adenosine in venous blood collected from normal, healthy subjects [419] can theoretically bind to 1.5–5 nM of plasma melatonin. However, the lower range of 1.5 nM already reflects the highest 1.13 nM median level detected in nocturnal plasma melatonin concentration in children between the ages of 1–3 [420], and melatonin production begins to decline at the early age of 20–30 to approximately 0.12 nM after the age of 50 [421,422,423].

Furthermore, although there are contrary outcomes in some other reports, there may be conditions where endogenous production of melatonin is suppressed by constant exposure to 60 Hz magnetic field [424] and ambient light at night [425,426]. In addition to binding adenosine, melatonin can significantly elevate ATP production in mitochondria [347,348]. Therefore, the adenosine moiety effect of ATP in phase separation is directly affected by how much melatonin is available, and the dosage of melatonin becomes a critical moving target in the study of phase separation regulation in dementia.

## 4. Of Mice and Men: Perfecting the Human Equivalent Dose for Melatonin in the Regulation of Phase Separation in Dementia

The in vitro and in vivo effects of melatonin in dementia is not only dose-dependent, but may also be time-, and perhaps even age-dependent (Table 2). Three experiments that tested the same strain of transgenic Tg2576 AD mice with 0.016, 0.5, and 2.0 mg/mL of melatonin added to the drinking water starting at various ages, produced not only different, but also contradictory results (Table 2). Tg2576 mice are leaner compared to wild types as they age [430,431,432]. Assuming an average weight of 22.5 g for each animal drinking 3 mL of water per day [361,362], the approximate daily melatonin supplementation would have been 2.13, 66.66, and 266.66 mg/kg, respectively.

Tg2576 mice receiving ~2.13 mg/kg daily starting at age 14 months failed to show any benefit in the reduction in Aβ accumulation in the brain or oxidative stress levels [363]; whereas Tg2576 mice receiving ~66.66 mg/kg daily starting at age 4 months showed a significant reduction in Aβ levels in brain tissues, as well as lowered abnormal nitration of proteins [362]. Importantly, Tg2576 mice receiving ~266.66 mg/kg daily starting at age 4 months produced the most impressive results where the brains of mice terminated at 15.5 months not only exhibited a dramatic decline in oligomeric Aβ40, but also a significant increase in soluble monomeric Aβ40. A noticeable decreasing trend in Aβ42 was observed in treated compared to untreated mice at the same age [361]. When Tg2576 mice from two separate experiments were administered ~266.66 mg/kg melatonin in drinking water daily starting at age 4 months until termination at 15.5 months, survival was significantly increased in treated compared to untreated mice [361,362]. Melatonin treatment at ~266.66 mg/kg daily in drinking water was able to reduce mortality in Tg2576 mice to levels observed in wild-type mice [361] (Table 2). Consequently, the effective translation of melatonin doses between animals and humans becomes the primary consideration when designing the dosage for clinical trials.

### 4.1. Aiming at Moving Targets in Allometric Scaling of Melatonin Interspecies Conversion

Animals have different metabolic rates. In general, larger animals have lower metabolic rates; therefore, the metabolic rate requires scaling in the conversion of interspecies doses. Allometry broadly describes the study of consequences between body and organ sizes [433,434]. The concept of interspecies allometric scaling was first presented in 1637 by Galileo Galilei [435]. Since that time, various allometric approaches have been proposed and used to determine the most efficacious human equivalent dose (HED) [436,437,438,439,440,441,442,443,444,445,446,447,448,449,450]. However, the identification of a definitive unified principle that effectively scales and optimizes different energy metabolism systems across animal species remains elusive and highly controversial [441,451,452,453,454].

In 1880, Rubner first proposed the body surface law that scales metabolic rate with body mass raised to the power of ⅔ [455]. The seminal work by Kleiber in 1932 led to extensive empirical evidence that supports the metabolic rate in most animals and plants scales to the power of ¾ of body mass instead of ⅔ [454,456]. To date, there is no consensus as to whether ⅔ or ¾ power of body mass should be used as the basal metabolic rate scale in connection with the body mass to determine dose conversion. In general, the exponent of ⅔ may be more applicable for pharmaceuticals that are eliminated via the kidneys, whereas the exponent of ¾ is more suitable for molecules such as melatonin that are cleared by metabolism or via combined metabolism and renal elimination [441,445]. However, once a correction factor (K_m_)—a ratio that accounts for the interspecies difference between humans and animals obtained by dividing body weight by body surface area (BSA) [447,457]—is applied during the conversion of animal to human doses, where

**HED (mg/kg) = Animal dose (mg/kg) × (animal K_m_/human K_m_)**

The results obtained are invariably higher than the ⅔ metabolic scale exponent of 0.67 (assuming human K_m_ = 37.9).

For example, to calculate the HED for a 70 kg human, BSA 1.846, K_m_ 37.9, the metabolic rate exponents for a mouse weighing 0.02 kg, BSA 0.007 (M^2^), K_m_ 2.857, and K_m_ ratio 13.265; and a rat weighing 0.15 kg, BSA 0.025 (M^2^), K_m_ 6, and K_m_ ratio 6.317, after adjustments would be 0.683 and 0.700, respectively. For a mini pig weighing 40 kg, BSA 1.14 (M^2^), K_m_ 35.088, and K_m_ ratio 1.08, the exponent for metabolic scaling for a 70 kg human becomes 0.863.

Thus, the accuracy of HED calculations during interspecies conversion is strictly dependent upon both body weight and BSA. The human BSA can be estimated via the formula BSA = ⅙ (Weight × Height)^0.5^ [458], whereas specific animal BSA is often more difficult to ascertain. Popular manuals containing instructions for interspecies HED conversions rely upon predetermined animal BSAs based upon a mathematical formula (BSA = *k*W^2/3^) postulated by Meeh in 1879, where BSA is derived from a constant *k* and volume estimated from body mass that is scaled to the ⅔-power. For most small animals including mice, the mean constant *k* is accepted to be 9.83 [459,460]. However, empirical determination for Meeh constants in mice with different body compositions and shapes revealed a range from 9.822 (normal) to 8.288 (obese) [459]. Therefore, the difference in Meeh constants between measured and calculated values would be important considerations when using mice with altered body composition. Furthermore, modifications for cell porousness per fractal theory indicate that the scaling exponent can vary from 0.694 to 0.83 [450].

As such, the estimated HEDs in this review will be calculated employing metabolic rate scaling using body weight raised to the ¾-power where

**HED (mg/kg) = animal dose (mg/kg) × (WEIGHT[kg]_animal_/WEIGHT[kg]_human_)^(1−0.75)^**

Using this formula, the estimated HED for a rat weighing 0.2 kg with a 10 mg/kg melatonin dose will be 2.12 mg/kg and 2.4 mg/kg for a 100 kg and 60 kg human, respectively. Nevertheless, the often-large differential in interspecies bioavailability and pharmacokinetics that can be modulated by route of administration, dosage, solubility, and formulation must also be taken into account for the accurate determination of an efficacious HED during conversion/scaling processes.

### 4.2. The Many Faces of Bioavailability in the Interspecies Conversion of Melatonin

The bioavailability of a substance is generally accepted as a key pharmacokinetic parameter, expressed as a percentage, that describes the rate and extent the substance becomes available in the general circulation after being delivered from a pharmaceutical form. Absolute bioavailability is a percentage obtained by comparing extravascular administration to intravenous injection (IV) assumed to be 100% available, whereas relative bioavailability compares different routes or formulations without reference to an IV administration [461]. Early work found healthy male subjects who ingested a single 80 mg melatonin (gelatin capsules) displayed varying levels of peak serum melatonin and absorption levels (up to 25-fold difference) 60–150 min after ingestion. This peak level could be extended from ~1.5 h to 4–6 h when subjects were given one 80-mg capsule/h over a 3 h period [462]. Melatonin bioavailability in humans, absolute or relative, is delivery-, dose-, solubility-, and formulation-dependent.

#### 4.2.1. Administration Routes Modulate Melatonin Bioavailability

The bioavailability of melatonin is affected by different routes of administration, where the mean bioavailability for 25 mg of melatonin delivered via intravesical, transdermal, rectal, and vaginal administration in healthy female volunteers were 3.6%, 10.0%, 36.0%, and 97.8%, respectively, compared to IV administration [463]. However, the determination of the bioavailability of oral melatonin may be complicated by melatonin metabolism. In humans, melatonin is mainly cleared by first-pass hepatic metabolism. When the clearance of an IV melatonin dose was combined with plasma concentrations of oral doses from previous data, the calculated oral bioavailability of melatonin was estimated to be 3–6% after a 2.5 mg dose, 3–76% after an 80 mg dose, but only 9% after a 100 mg dose [464].

In 2000, DeMuro and coworkers determined the absolute oral bioavailability of 2 and 4 mg melatonin doses (tablets) to be 14.3% ± 7% and 15.9% ± 6%, respectively, compared to IV melatonin (2 mg) [465]. Fifteen years later, a systematic review of 22 studies identified from 392 records that tested oral or IV melatonin dosages between 0.3 and 100 mg found the bioavailability of melatonin to be approximately 15% with significant variability between individuals, where critically ill patients often displayed accelerated absorption but compromised elimination [466].

#### 4.2.2. Melatonin Bioavailability Is Inversely Correlated to 6-Sulfatoxymelatonin

As such, the interpretation of bioavailability may not be straightforward considering the fact that absolute bioavailability of oral melatonin has also been reported at ~3% (10 mg gelatin capsule) albeit with considerable variability among the 12 tested healthy male subjects (20–40 yr old) [467]. Low absolute bioavailability in oral melatonin is often the prominent effect of first-pass hepatic metabolism which produces the major melatonin metabolite 6-sulfatoxymelatonin (6-OHMS). Consequently, low endogenous production of melatonin in the elderly is associated with a significant reduction of 6-OHMS in older test subjects compared to younger ones (82–21 years old) [421]. However, a small study sample found a significant inverse correlation between oral bioavailability and 6-OHMS, where lower oral bioavailability (10%, 12%) was correlated with high plasma 6-OHMS (31%, 14%). Conversely, high bioavailability (56%, 54%) was associated with lower 6-OHMS in plasma (4%, 3%) of healthy male subjects (21 to 32 years old) tested [468].

This inverse relationship was also observed in children admitted to an intensive care unit where septic patients who did not survive exhibited nocturnal melatonin levels that were significantly higher than survivors, but total 6-OHMS excretion was dramatically lower in nonsurvivors compared to survivors. Additionally, septic shock patients had higher nocturnal melatonin levels than non-septic patients [469]. Low plasma 6-OHMS is correlated with autism [470,471], and low 6-OHMS excretion level is associated with adults who were lean at birth but obese in adult life, and high excretion rates were associated with opposite observations [472]. Similarly, patients with unstable angina exhibited significantly lower 6-OHMS than healthy controls and no negative correlation with age was observed in coronary patients as opposed to healthy subjects [473]. Therefore, the interpretation of melatonin bioavailability becomes more meaningful when 6-OHMS levels are taken into consideration.

#### 4.2.3. Animals Show Large Variations in Melatonin Bioavailability

In animals, melatonin bioavailability via different administration routes varies greatly with strain, species, and first pass metabolism after administration. Yeleswaram and coworkers determined the absolute bioavailability of melatonin for a 10 mg/kg oral dose compared to IV in male Sprague Dawley (SD) rats to be 53.5%, but more than 100% in dogs and monkeys. However, the oral bioavailability in dogs is dose-dependent, where 1 mg/kg resulted in only 16.9% bioavailability. IP injection of melatonin at 10 mg/kg in SD rats increased bioavailability to 74.0% compared to oral at 53.5%.

In rats, IV administration at half the dose (5 mg/kg) achieved 80% bioavailability via IP at 10 mg/kg [474]. Rats, regardless of strain and administration, metabolize melatonin completely. SD rats excreted 60–70% of radiolabeled melatonin via IV injection as the major metabolite 6-OHMS [475]; and male Wistar rats administered 12.5–250 µg melatonin via IP injection also showed concentration-dependent increases in plasma of melatonin and 6-hydroxymelatonin, which always maintained a constant ratio of 1% of plasma melatonin irrespective of dosage. However, the sulfate conjugate 6-OHMS reached at maximum, ~64-fold elevation of maximum plasma 6-hydroxymelatonin levels [476].

Similar to rats, female C57BL/6 mice (age 8–10 weeks) administered varying doses of melatonin at 31.25, 62.5, 125, 250, and 500 mg/kg showed no difference in the ability to clear and eliminate melatonin; and the concentration of melatonin in the liver and gastrointestinal tracts was higher than other vital organs by 5- to 10-fold, indicating that hepatic first-pass metabolism is also prominent in mice. However, the effect of melatonin in mice is also dose-dependent even at supra-pharmacological concentrations. After exposure to lethal radiation, mice administered 500 mg/kg had the highest survival rate (55%) compared to 250 and 125 mg/kg (40%) [477].

#### 4.2.4. Solubility and Formulation Modulate Melatonin Bioavailability

The oral bioavailability of melatonin, at any dose, can be modulated by altering solubility. The oral bioavailability of melatonin in critically ill patients with sepsis was greatly enhanced by the use of solvents, where melatonin dissolved in glycerol achieved a 5-fold increase in relative bioavailability over melatonin in capsules at the same doses (20 or 50 mg) [478]. Similarly, when compared to IV solution (62.5 mg/kg dissolved in water), absolute oral bioavailability in mice of an aqueous melatonin suspension at 250 mg/kg administered via gavage tube was 29%, whereas 250 mg/kg melatonin dissolved in a popular co-solvent polyethylene glycol 400 (PEG400) and administered in the same manner achieved absolute bioavailability of 98.5% [477]. However, PEGs are very hydrophilic molecular crowders that can amplify entropy gain from water-release, causing dehydration that drives phase separation [136]. Hence, the use of PEG as a solvent in applications for the regulation of phase separation must be carefully weighed.

Variations in formulation also affect melatonin bioavailability. In rabbits, intranasal delivery of melatonin encapsulated in starch microspheres achieved absolute bioavailability of 84.07%, whereas intranasal administration via solution produced much lower pharmacokinetics [479]. While intranasal melatonin administration in male Wistar rats via niosomes—bilayer vesicles of nonionic surfactant-based liposomes—achieved absolute bioavailability of 98.7% compared to IV melatonin solution [480]. Therefore, the successful conversion of an animal melatonin dose into an efficacious human equivalent requires equal considerations of metabolic rate scaling, bioavailability as determined by intrinsic differences between species, administration route, as well as solubility and formulation.

### 4.3. Timing Is Everything in the Dosing of Melatonin for the Regulation of Phase Separation in Dementia

The daily supra-pharmacological dose of 266.66 mg/kg administered in drinking water to transgenic Tg2576 mice from 4 months to 15.5 months not only prevented aggregation of amyloid fibrils but also prolonged survival compared to untreated mice [361,362]. This dose can be converted into a HED using metabolic scaling to the ¾-power, with the assumption of mice and human body weight to be 0.0225 kg and 70 kg, respectively; and oral bioavailability of mice and humans to be 63.75% and 15%, respectively. 63.75% oral bioavailability is a conservative estimation of a 50% enhancement of solubility in water achieved by first dissolving melatonin in hydroxy methyl cyclodextrin before dilution in drinking water to the final concentration of 2 mg/mL [361]. Cyclodextrins (CDs) are small carbohydrates that enhance the solubility of molecules and drugs, resulting in higher bioavailability [481,482]. The HED obtained before the bioavailability adjustment is 2499 mg. Without solubility enhancement, the adjusted bioavailability HED dose is 4831 mg. After correcting for a 50% enhancement in bioavailability (calculated based on oral bioavailability data obtained by Choudhary et al. [477]), the final HED is a staggering dose of 10,621 mg. 

Even though the toxicity of melatonin as defined by LD_50_ has not been determined in human or rodents, where early studies failed to produce death in mice at 800 mg/kg [483], and acute oral toxicity that result in LD_50_ in rats is reported at concentrations higher than 3200 mg/kg (in one single dose [484]) according to the latest Merck safety data sheet on melatonin (Regulation (EC) No. 1907/2006, revised 17 November 2021), without a convincing rationale for a high HED in the context of phase separation in dementia, this extreme supra-pharmacological HED may seem unjustified.

#### 4.3.1. The Rationale for Frequent Division of Melatonin Doses

The Tg2576 mice drank ~3 mL of water containing a total of 6 mg melatonin in a 24 h period. Accordingly, total HED should also be administered in divided doses of 885 mg × 12. This hypothetical HED now resembles the HED used by Martin et al. to elevate ATP production via complex I and COX (complex IV) activities in the brain and liver mitochondria of rats [348].

Male Wistar rats with a body weight between 200–230 g were administered 10 mg/kg melatonin via IP injection. Respiratory complex activity enhancements were tissue- and time-dependent. Complex I activities in the liver achieved peak levels and returned close to baseline at ~30 and ~180 min, respectively; whereas in the brain, peak activity levels were attained at ~60 min and returned to baseline at ~180 min. COX activities in both the liver and brain became significantly elevated at ~30 min, but reached a peak in the liver at ~100 min before declining close to baseline at 180 min, whereas brain activities quickly dropped to baseline soon after 120 min [348]. In other words, in the brain, complex I and COX reached peak activity levels at 60 and 30 min, respectively, before returning to baseline at ~120 min. Whereas in the liver, complex I and COX activities were both elevated at ~30. Complex I steadily declined to close to baseline at ~180 min, but COX activities remained elevated and reached a peak at ~100 min before declining close to baseline at ~180 min.

The difference in peak and duration of respiratory enzyme activities may reflect the effect of prominent first-pass hepatic metabolism where more melatonin is retained in the liver and gastrointestinal tracts than other vital organs such as the brain [475,477]. Regardless, bioavailability via IP in rats is ~74%, or 4.933-fold higher than oral bioavailability in humans. Therefore, assuming an average body weight of 0.215 kg and 70 kg for rats and humans, respectively, the total daily HED that can effectively maintain peak complex I and COX activity at a sustained level throughout a 24 h period in order to provide adequate ATP and adenosine that can prevent and solubilize aberrant phase separation and aggregation is 9755 mg, adjusted for differences in metabolic rate and bioavailability, assuming average intake of 812.90 mg × 12 in a 24 h period. However, the amount quickly doubles to 19,509.60 mg if maximum complex I and COX activities were to be sustained in the brain over a 24 h period based on observations reported by Martin et al. [348].

**Figure 3:**
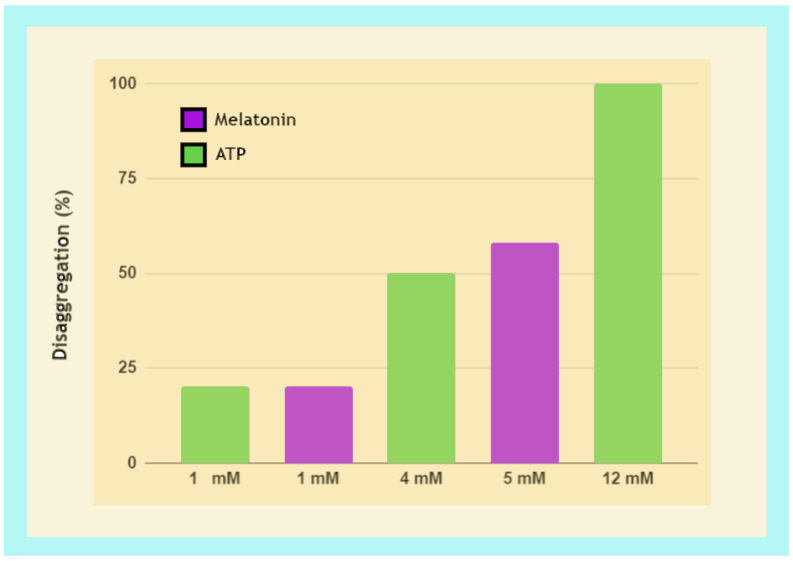
Comparison of disaggregation percentages obtained when different concentrations of melatonin and ATP were added to mediums containing repeat tau aggregates [367] and egg white aggregates [388], respectively.

At this point, the estimated daily total HED of 10,621 mg obtained from Tg2576 mice taking 266.6 mg/kg in drinking water becomes quite reasonable and theoretically justifiable. In addition, in vitro work found a strong correlation between ATP and melatonin concentration for disaggregation of fibrils [367,388], where 1 mM of ATP and melatonin both were able to dissolve 20% of aggregates, respectively; 4 mM and 5 mM of ATP and melatonin dissolved 50% and ~60% of aggregates, respectively (Figure 3). Therefore, the rationale supporting supra-pharmacological oral melatonin doses to maintain elevated ATP synthesis that prevents aberrant phase separation and aggregation warrants further investigation.

#### 4.3.2. The Calculation of HED Estimates Adjusted for Differences in Metabolic Rates, Bioavailability, and Formulation

A close examination and comparison of various HED estimates obtained from the different in vivo experiments discussed in Table 2 may provide clarification on melatonin doses required for the effective regulation of phase separation in dementia. Importantly, there is a difference in doses required to obtain similar results in healthy versus diseased, transgenic animal models.

Table 3 illustrates how (A) oral melatonin HED for a human weighing 70 kg is converted from animal doses by using metabolic rate scaled to ¾ power with body weight (M_b_^3/4^); (B) where HED (A) is further adjusted by interspecies bioavailability difference that takes into account both species differentials and administration routes; and (C) adjusts (A) to reflect enhancements via solubility/formulation as per study design. In the absence of data, where applicable, the average body weight of transgenic Tg2576 and wild-type mice is assumed to be 0.0225 kg and 0.025 kg, respectively. Daily food intake for mice [364] is assumed to be ~4.5 g [432]. The oral bioavailability of melatonin in humans and mice is assumed to be 15% [465,466] and 29% [477], respectively, in the conversion for values in column (B). Bioavailability enhancement via increased solubility is estimated at a conservative 50% increase based on data reported by Choudhary and coworkers [477]. Therefore, values in column (C) are obtained by multiplying (A) by 4.25.

The selection of a “perfect” HED dose for melatonin under different contexts is ultimately at the sole discretion of the investigator(s) who will determine the “parameters to be scaled, independent variables, and the mathematical relationship used in the scaling process” [438,439]. Therefore, the values presented in Table 3 are intended purely as an informative guide to various potentially effective HEDs for melatonin that can be applied in the regulation of phase separation in dementia under distinct conditions.

## 5. Conclusions

Modernization of infrastructure leads to inevitable environmental changes that restrict easy access to the ancient, dynamic synergy between light, water, and melatonin. Individuals who live in densely populated urban areas are affected by the lack of adequate greenness that limits exposure to red and infrared frequencies from sunlight, generously reflected by plants [205]. Furthermore, continuous exposure to low-level microwaves and varying levels of EMF can restructure hydrogen bonding to either decrease or increase intracellular viscosity [195,196,197,198,199]. Even exposure to magnetic fields at 0.5 T causes water molecules to form new hydrogen bonds resulting in larger-sized water clusters that increase viscosity but reduce the proportion of free water molecules [485]. At the same time, the endogenous production of melatonin may be impacted under some circumstances by constant exposure to 60 Hz magnetic field [424] and ambient light at night [425,426]. In older adults with varying risks for dementia, increased light exposure in the evening results in earlier dim light melatonin onset (DLMO) time. This shift in the circadian phase may disturb rhythmicity that is often associated with dementia [486,487,488].

Our brave, new world offers unlimited potential in technological advances in every frontier imaginable but exacts an exorbitant premium on our health by creating intracellular conditions that favor aberrant phase separation resulting in pathological protein aggregations that are associated with a wide range of health challenges, including dementia. The reinstatement of this powerful but lost synergy is a provocative proposal that entails the conditional rescaling of an ancient theme to harmonize with the cacophony of modern influences, restoring, once again, balance in optimum health.

## Figures and Tables

**Table 1:** A sample collection of popular wavelengths employed in photobiomodulation, starting from visible 670 nm to non-visible near- and far-infrared wavelengths, and their effects on various symptoms associated with dementia in animals and humans.

Wavelength	Model/Cell Line/Device	Duration/Intensity	Results	Ref.
670 nm	APP/PS1 AD transgenic mice/transcranial LED	90 s (4 Joule/cm^2^)/day × 20 over 4 wks	Attenuated cerebellar cortex Aβ deposition, fibril formation.	[207]
670 nm	K3 tau, APP/PS1 AD transgenic mice/transcranial LED	90 s (4 Joule/cm^2^)/day × 20 over 4 wks	Neocortex and hippocampus of K3 and APP/PS1 mice showed reduction in tau/fibril formation and size/number of Aβ, respectively.	[208]
670 nm	C57BL/6, transgenic 2576 mice/transcranial LED	90 s (4 Joule/cm^2^)/day × 20 over 4 wks	All mice showed reduced Aβ oligomer binding at CNS synapses.	[209]
670 nm	h tau, 3xTgAD mice/transcranial LED	90 s (4 Joule/cm^2^)/day × 20 over 4 wks	Reduced toxic tau oligomers, improved memory deficits, upregulated clearance of misfolded proteins in both models	[210]
808 nm	Aβ-treated microglia cells from health mice/diode laser	5 min (9 Joule/cm^2^)	Exceeded control cell ATP production after 24 h by 155%, suppressed ROS production promoting neuronal survival.	[211]
810 nm	8 patients diagnosed with dementia/transcranial+transnasal LED	20 min (pulsed at 40 Hz at 50% duty cycle), 3 times/wk for 12 consecutive wks	Significant score improvements in ADAS-cog (13.8%) NPI-FS (61.4%) compared to baseline ^1^.	[212]
1060–1080 nm	11 patients with dementia/transcranial LED helmet	6 min (1100 LEDs pulsed at 10 Hz at 50% duty cycle)/day × 28 consecutive days	Improved executive functioning in clock drawing, immediate recall, praxis memory, visual attention, and task switching.	[213]
1060–1080 nm	60 patients with mild to moderate dementia/transcranial LED helmet	2 × 6 min (23.1 mW/cm^2^)/day × 8 consecutive weeks	Improved cognitive functions, auditory and verbal learning, processing speed, mood, energy, and sleep.	[214]
1060 nm	27 healthy participants aged 45+/transcranial LED helmet	2 × 6 min (12 mW/cm^2^)/day × 28 minimum	Significant improvements in motor function, memory performance, and processing speed.	[215]
1040–1090 nm	APP/PS1 AD double-transgenic mice/LED irradiation	6 min (15 mW/cm^2^)/day × 55 with a 28-day suspension after day 40	Improvement in memory, spatial learning ability, and modest plaque reduction; suspension period indicated treatment effects were transient.	[216]
500 nm/800 nm/3–25 µm	APP/PS1 AD double-transgenic mice/LED irradiation	60 min (0.13 mW/cm^2^)/day × 45	FIR (3–25 µm) enhanced Aβ phagocytosis via increased ATP production and attenuated cognitive dysfunction compared to other wavelengths tested.	[217]

^1^ ADAS-cog Alzheimer’s Disease Assessment Scale-cognitive; NPI-FS: Neuropsychiatric Inventory frequency severity.

**Table 2:** In vitro and in vivo studies that reveal important relationships between melatonin dosage, timing, and administration that produced different results for symptoms associated with dementia.

Melatonin Dosage/Duration	Study Design	Results	Ref.
25 µM, 250 µM, 2.5 mM	In vitro α-Synuclein peptide aggregation	Blocked α-Syn fibril formation and destabilized preformed fibrils in a dose- and time-dependent manner; increased viability of primary mixed neurons treated with α-Syn to ~97% in a time-dependent manner.	[356]
10 mg/kg (IP) × 5/day for 2 days, then × 2/day for 5 days	Arsenite-induced oxidative injury in substantial nigra of adult male rats	Attenuated arsenite-induced α-Syn aggregation, lipid peroxidation, and glutathione depletion.	[357]
100 µM melatonin	Aβ peptides (1-40) and (1-42) β-sheet/fibril formation	Progressive reduction in Aβ1-40 β-sheet structures to 24% after 24 h incubation; immediate reduction in Aβ1-42 β-sheet structures from 89% to 65%, decreasing to 59% after 4 h.	[358]
Melatonin dissolved in 2 mM ammonium acetate	Aβ peptide (1-40) β-sheet/fibril formation	Inhibited β-sheet formation by targeting hydrophobic Aβ-peptide segment (29-40) intermolecular activities.	[359]
1 mM melatonin	Aβ1-40 peptide, profibrillogenic apoE4/apoE	Melatonin alone delayed fibril formation from 24 h up to 72 h. Combined with either apoE4 or apoE3, inhibition remained effective at termination of experiment.	[360]
2 mg/mL in drinking water starting at age 4 months until euthanasia	Transgenic Tg2576 AD mice, terminated at 4 months 1 wk or 15.5 months	The brains of animals treated with melatonin terminated at 15.5 months exhibited dramatic decline in oligomeric Aβ40 together with a significant increase in soluble monomeric Aβ40, and a decreasing trend in Aβ42 compared to untreated mice at same age. Melatonin prolonged survival rates of 15.5-month mice to levels attained by non-transgenic mice.	[361]
2 mg/mL in drinking water starting at age 4 months until euthanasia at 15.5 months	Transgenic Tg2576 AD mice	Increased survival in treated mice (3 deaths/41 survivals) compared to untreated (13 deaths/31 survivals).	[362]
0.5 mg/mL in drinking water starting at age 4 months	Transgenic Tg2576 AD mice	Striking reductions in Aβ levels in brain tissues of treated mice at 8, 9.5, 11, and 15.5 months.	[362]
16 µg/mL in drinking water starting at age 14 months	Transgenic Tg2576 AD mice	Melatonin treatment failed to reduce brain Aβ levels or even oxidative damage.	[363]
40-ppm (*w/w*) in pelleted minimal basal diet	Male B6C3F1 mice aged 6, 12, and 27 months	Significant reduction in Aβ in brain cortex tissues: 57% in Aβ40 and 73% in Aβ42; increased melatonin levels in cerebral cortex in all 3 treated age groups (12 > 6 > 27 mos) compared to untreated.	[364]
10 mg/kg (IP) daily for 3 weeks	Male wild-type C57BL/6N mice (8 wks old) injected with Aβ1-42 peptide	Melatonin treatment reversed Aβ1-42-induced synaptic disorder, memory deficit, and prevented Aβ1-42-induced apoptosis, neurodegeneration, and tau phosphorylation.	[365]
10 mg/kg in drinking water from day 7 after tauopathy induction to day 28 at termination	4-month-old C57BL/6J mice injected with human tau mutation P301L (AAV-hTau)	Increased ROS and tau hyperphosphorylation starting at day 7 precedes cognitive decline; melatonin-treated animals showed reduced memory impairment, tau hyperphosphorylation, ROS, and neuroinflammation.	[366]
10 μmol/L	Ex vivo brain slices from 3-month-old SD rats exposed to okadaic acid to induce tau hyperphosphorylation	Melatonin reduced tau hyperphosphorylation and ROS to control levels in OA-treated brain slices.	[366]
100 μM–5000 μM	Aggregation/disaggregation of repeat domain Tau (K18wt)	Pre-formed tau fibril disaggregation was dose-dependent: 14% with 100 μM, 54% with 5000 μM.	[367]
200–5000 μM	Aggregation/Disaggregation full-length tau (hTau40wt)	Tau treated with 200 μM melatonin showed no change in morphology compared to controls; 5000 μM melatonin treatment did not prevent aggregation but disaggregated tau fibrils into broken filaments.	[368]

**Table 3:** Calculations of three HEDs converted from animal doses using different adjustments that account for differences in (A) Metabolic rates by scaling to the ¾-power; (B) Bioavailability; (C) Bioavailability that is enhanced by solubility and/or formulation.

Study Design/Total Daily Dose/Duration/Ref.	Results	(A) HED Daily Total (mg/kg) Scaled to M_b_^3/4^	(B) Dose (A) Adjusted by Bioavailability	(C) Dose (A) Adjusted by Enhanced Bioavailability
2 mg/mL in drinking water, Tg2576 AD mice/266.66 mg/kg/11.5 mos starting at 4 mos old/[361,362]	Striking reductions in Aβ aggregates at all ages during treatment; dramatic extension of survival of AD mice to levels similar to wild types.	2499 mg (35.7 mg/kg)	4831 mg (69 mg/kg)	10,621 mg (151.73 mg/kg)
0.5 mg/mL in drinking water, Tg2576 AD mice/66.66 mg/kg/11.5 mos starting at 4 mos old/[362]	Striking reductions in Aβ levels in brain tissues of treated mice at 8, 9.5, 11, and 15.5 months.	625 mg (8.928 mg/kg)	1208 mg (17.26 mg/kg)	2656 mg (37.94 mg/kg)
0.016 mg/mL in drinking water, Tg2576 AD mice/2.13 mg/kg/10 wks starting at age 14 mos old/[363]	Failed to reduce brain Aβ levels, unable to reverse oxidative damage.	19.96 mg (0.285 mg/kg)	38.58 mg (0.55 mg/kg)	84.83 mg (1.21 mg/kg)
10 mg/kg in drinking water, healthy, normal C57BL/6J mice/14 days after tauopathy initiation/[366]	Reduced memory impairment, tau hyperphosphorylation, ROS, and neuroinflammation.	96.23 mg (1.375 mg/kg)	186.0 mg (2.66 mg/kg)	408.98 mg (5.84 mg/kg)
40 ppm in food pellets, healthy, normal B6C3F1 mice/7.2 mg/kg/11 weeks different age groups/[364]	Significant reduction in Aβ peptides in brain cortex tissues: 57% in Aβ40 and 73% in Aβ42; increased melatonin levels in cerebral cortex in all 3 treated age groups (12 > 6 > 27 mos) compared to untreated.	69.29 mg (0.99 mg/kg)	133.94 mg (1.91 mg/kg)	Not applicable
10 mg/kg IP injection, C57BL/6J mice treated with Aβ1-42 peptide/daily IP injections for 3 wks/[365]	Reversed Aβ1-42-induced synaptic disorder, memory deficit; prevented Aβ1-42-induced apoptosis, neurodegeneration, and tau phosphorylation.	98.55 mg (1.41 mg/kg)	486.15 mg (6.95 mg/kg)	Not applicable

## Data Availability

Not applicable.

## Abbreviations

6-OHMS	6-sulfatoxymelatonin
Aβ	amyloid-β
AD	Alzhemer’s disease
AMP	adenosine monophosphate
α-syn	alpha-synuclein
ATP	adenosine triphosphate
CP	centipoise
COX	cytochrome C oxidase
EMF	electromagnetic fields
FTD	frontotemporal dementia
FUS	fused in sarcoma
H_2_O_2_	hydrogen peroxide
IDP	intrinsically disordered protein
IP	intraperitoneal
IV	intravenous
LCD	low-complexity domain
MD	molecular dynamics
MLO	membraneless organelle
^•^OH	hydroxyl radical
PD	Parkinson’s disease
pI	isoelectric point
ROS	reactive oxygen species
SD	Sprague Dawley
SG	stress granule
VaD	Vascular dementia
